# Rutin protects against gamma-irradiation and malathion-induced oxidative stress and inflammation through regulation of mir-129-3p, mir-200C-3p, and mir-210 gene expressions in rats’ kidney

**DOI:** 10.1007/s11356-023-27166-z

**Published:** 2023-05-15

**Authors:** Amel F. M. Ismail, Asmaa A. Salem, Mamdouh M. T. Eassawy

**Affiliations:** 1 Drug Radiation Research Department, National Center for Radiation Research and Technology (NCRRT), Egyptian Atomic Energy Authority (EAEA), Cairo, Egypt; 2 Regional Center for Food and Feed (RCFF), Agricultural Research Center, Giza, Egypt

**Keywords:** Nephroprotection, Radioprotection, Rutin, Malathion, Gamma irradiation, Kidney injury, Micro-RNA, Rats

## Abstract

Kidney injury represents a global concern, leading to chronic kidney disease. The organophosphate insecticide malathion (MT) demonstrates environmental disturbance and impairment of different mammalian organs, including kidneys. Likewise, gamma-irradiation (IRR) provokes destructive effects in the kidneys. Rutin is a flavonoid glycoside that exhibits nephro-protective and radio-protective properties. This manuscript focused on investigating the protective response of rutin on MT- and IRR-triggered kidney injury in rats. Rats were randomly divided into eight groups of twelve: G1 (C), control; G2 (Rutin), rutin-treated rats; G3 (IRR), gamma-irradiated rats; G4 (MT), malathion-treated rats; G5 (IRR/MT), gamma-irradiated rats treated with malathion; G6 (IRR/Rutin), gamma-irradiated rats treated with rutin; G7 (MT/Rutin), rats treated with malathion and rutin; and G8 (IRR/MT/Rutin), gamma-irradiated rats treated with malathion and rutin, every day for 30 days. The results demonstrated that rutin treatment regulated the biochemical parameters, the oxidative stress, the antioxidant status, and the inflammatory responses due to the down-regulation of the renal NF-κB p65 protein expression. Moreover, it amended the activity of acetylcholinesterase (AchE), angiotensin ACE I, and ACE II-converting enzymes. Besides, it regulated the iNOS, eNOS, miR-129-3p, miR-200c, and miR-210 gene expressions and bradykinin receptor (B1R and B2R) protein expressions. Histopathological examinations of the kidney tissue confirmed these investigated results. It could be concluded that rutin demonstrated nephro/radioprotection and counteracted the toxicological effects triggered in the kidney tissues of IRR, MT, and IRR/MT intoxicated rats, via regulating miR-129-3p, miR-200c-3p, and miR-210-3p gene expressions, which consequently regulated B2R protein expressions, ACE II activity, and HIF-1α production, respectively.

**Supplementary Information:**

The online version contains supplementary material available at 10.1007/s11356-023-27166-z.

## Introduction

Exposure to chemical or physical pollutants instigates nephrotoxicity, which can form a global concern due to inattention of the disease diagnoses and management, leading to chronic kidney disease (CKD). CKD is associated with diabetic and cardiovascular complications (Salem and Ismail 2021). The manipulation of the organophosphate insecticide malathion (*O*,*O*-dimethyl S-(1, 2-dicarbethoxyethyl, MT) is not limited to controlling pests in agricultural sectors, but also handled indoors and outdoors to control pets, and provide livestock protection. As a result, malathion deposits can precipitate in soils, plants, and foodstuffs that can easily be transferred and encourage the environmental disturbance and impairment of different human beings’ organs (Alex and Mukherjee 2021; Nangare et al. 2022). Humans acquire several toxicological problems that have resulted from direct exposure or indirect administration of malathion that is mainly due to the repression of acetylcholinesterase activity (Ince et al. 2017). Malathion toxicity extended to affect all body organs, including the kidneys (Selmi et al. 2018; Fenik et al. 2011; Poomagal et al. 2021). Acute and chronic exposure to malathion convinces oxidative stress in vivo and in vitro, leading to depression of the antioxidant protective system, DNA damage, and impairment of the kidney functions (Ince et al. 2017), leading to chronic kidney diseases (CKD), which may terminate with kidney failure. Malathion also exhibited immune-toxicity and inflammatory effects that should be organized (Hefnawy et al. 2016). The effect of malathion on kidney impairment has not been appropriately studied in the scientific research, which requires further investigations.

Ionizing radiation has evoked a lot of consideration due to its benefits in medical, agricultural, and industrial fields, as well as its achievable destructive effects on the human population. The deleterious effects of ionizing radiation have been contributed to the generation of reactive oxygen species (ROS), through water radiolysis. Radiation reduces the activity of endogenous antioxidant enzymes, which are the first line of defense in maintaining redox balance and normal metabolic activities. ROS can instigate DNA, protein, and lipid damage in cells by facilitating an imbalance of pro-oxidant and antioxidant status (Jit et al. 2022; Srinivasan et al. 2007). Antioxidant defenses, both enzymatic (as superoxide dismutase (SOD) and catalase (CAT)) and non-enzymatic (including glutathione (GSH)), counteract the damage caused by ROS. Antioxidants are produced by the human body or obtained through diet in normal circumstances, and they can lower the reactive species concentrations in both cases. The imbalance between the production of reactive species and the antioxidants designates oxidative stress, which affects different organs, including the kidneys (Mahgoub et al. 2020; Maurya et al. 2006). Kidneys are very sensitive organs toward radiation exposure, especially radiotherapy. Accordingly, natural pharmaceuticals, as pure natural compounds, or herbal remedies are under inquiry to protect or limit the hazardous impacts of radiation nephropathy (Turkyilmaz et al. 2022).

Rutin (quercetin 3 rutinoside or (3,3′,4′,5,7‐pentahydroxyflavone‐3‐rhamnoglucoside) is a naturally occurring flavonoid glycoside (the phenolic compound quercetin is the basic unit of rutin, which is substituted with glucose and rhamnose sugar groups at the hydroxy group in position C-3 (see supplementary data)) that is present in passionflower, buckwheat, spinach, leaf of tomato, apples, onions, and tea (Aksu et al. 2017). It belongs to the bioflavonoid family, and it has anti-carcinogenic, antioxidant, anti-viral, antiallergenic, anti-inflammatory, and anti-apoptotic effects (Nafees et al. 2015). These antioxidant and anti-inflammatory specifications of rutin counteracted different nephrotoxic symptoms in rat model (Diwan et al. 2017). Moreover, rutin demonstrated radio-protective properties (Sunada et al. 2014).

The effect of malathion and gamma-irradiation on kidney impairment has not been sufficiently investigated, which needs more exploration. In view of that, this manuscript aims to study the nephroprotective response of rutin, concerning the relations of the gene expression ratios of miR-129-3p, miR-200c, and miR-210, as well as the protein expression ratios of NF-κB p65 and bradykinin receptors (B1R and B2R) to the developed oxidative stress, and the emerged inflammation by IRR and MT exposure triggered kidney injury in rats.

## Materials and methods

### Chemicals

Malathion (Nasr lathion®, 1%WP) was provided by El-Nasr Chemical Industries Co., Egypt. Rutin, urethane, and carboxymethyl cellulose (CMC) were obtained from Sigma Aldrich. Formalin was obtained from El-Gomhouria Co., Egypt. Biodiagnostics kits (Cairo, Egypt) were used to determine oxidative stress parameters and antioxidant enzyme activity. Sodium and potassium levels were assessed by DiaSys Diagnostic Systems (GmbH, Germany) kits. ELISA kits for rats were obtained from MyBioSource and Elabscience (USA). Total RNA extraction kit was obtained from Qiagen (USA). Polyvinylidene fluoride (PVDF) membranes were obtained from Bio-Rad (Bio-Rad Laboratories, Inc. Life Science Research Group, CA, USA). TRIzol reagent, primary and secondary antibodies (phospho-nuclear factor kappa-light-chain enhancer of activated B cells p65 subunit (p-NF-κB p65, Ser536) monoclonal antibody (T.849.2, Cat: MA5-15,160), B1 bradykinin receptor rabbit polyclonal antibody (BDKRB1 (B1R), Cat. No: PA5-77,292, B2 bradykinin receptor rabbit polyclonal antibody (BDKRB2 (B2R), Cat No: 720288), β-actin (Cat. No: MA5-16,140), and TaqMan™ MicroRNA reverse transcription kit (Cat. No:4366597) were obtained from Thermo Fisher Scientific (USA). mirVana™ miRNA isolation kit (Cat. No:AM1560) was obtained from Life Technologies Corporation (USA). miRNA-129–1-3pmiRNA PCR kit (Cat. No: MBS8297717), miR-200c-3p miRNA PCR kit (Cat. No: MBS8297433), and miR-210-3p PCR kit (Cat. No: MBS8297441) were obtained from MyBioSource. All other chemicals used for this study were of analytical grade.

### Irradiation facilities

The ^137^Cesium Canadian Gamma Cell-40 located at the National Center for Radiation Research and Technology (NCRRT) was used for the whole-body gamma-irradiation of animals. The radiation dose rate was 0.666 rad/s, during the time of exposure to achieve a total dose of 3 Gy (Rezk and Abdel-Rahman 2013).

### Animals

Ninety-six Wistar male albino rats, 120–150 g, 3 months age, were transmitted from the Helwan farm animal house, VACSERA (The Holding Company for Biological Products and Vaccines), Helwan, Egypt into the NCRRT animal residence. The animals were acclimatized under the proper laboratory conditions of illumination, temperature, humidity, and feeding with ordinary laboratory pellet diet, 21% protein, then incessant ad libitum drinking water performing the international and NCRRT guidelines for animal experiments. The Ethical Committee of the NCRRT approved this study (21A/21).

### Experimental design

Rats were randomly divided into eight groups of twelve: group 1 (G1: control; C): the animals were treated orally with 1.0 mL of carboxymethyl cellulose (CMC, 0.5% w/v) solution as well as 0.2 mL of corn oil every day for 30 days. Group 2: (G2: Rutin) rutin-treated rats, the rats administered orally with 1.0 mL of rutin solution (100 mg rutin powder suspended in CMC (0.5% w/v) solution /kg body weight (b.wt) by intra-gastric gavage every day for 30 days (Diwan et al. 2017). Group 3 (G3: IRR): the rats were exposed to a single dose of gamma irradiation; 3 Gy on day zero of the experiment. Group 4 (G4: MT): the animals received malathion by intra-gastric gavage, prepared in 0.2 mL corn oil at a dose of 50 mg/kg b. wt (Baiomy et al. 2015) every day for 30 days. Group 5 (G5: IRR/MT): the rats were exposed to a single dose of 3 Gy gamma-irradiation on day 0 of the experiment, then, after 24 h, the rats started MT oral administration (50 mg/kg b.wt in 0.2 mL corn oil) every day for 30 days. Group 6 (G6: IRR/Rutin): the rats were exposed to a single dose of gamma-irradiation; 3 Gy of the whole-body, 24 h before starting rutin administration (100 mg rutin powder suspended in CMC (0.5% w/v) solution/kg b.wt, every day for 30 days). Group 7 (G7: MT/rutin): the rats were orally administered with 1.0 mL of rutin solution (100 mg rutin powder suspended in CMC (0.5% w/v) solution/kg b.wt, 2 h before receiving MT (50 mg/kg b. wt in 0.2 mL corn oil) every day for 30 days. Group 8 (G8: IRR/MT/Rutin): the rats were exposed to a single dose; 3 Gy of whole body, after 24 h, rats were orally administered with 1.0 mL of rutin solution (100 mg rutin powder suspended in CMC (0.5% w/v) solution/kg b.wt, 2 h before receiving MT (50 mg/kg b. wt in 0.2 mL corn oil) every day for 30 days.

After 30 days of malathion and rutin administration, the rats spent an overnight fasting period. Then, the cardiac blood was collected from all animals in plain tubes under urethane anesthesia (1.2 g/kg b.wt.) (Moheban et al. 2016). The collected blood stood at 37 °C and centrifuged at 3000 g to separate the serum for the biochemical examination. The kidneys from each rat were immediately excised. Kidney tissue samples were collected and fixed in a 10% neutral buffered formalin solution for histopathological explorations. Other kidney samples were rinsed in ice-cold physiological saline, dried carefully, weighed, and stored at − 80 °C for subsequent analyses. For trace elements analysis, the kidney samples were rinsed in ice-cold de-ionized water, dried carefully, weighed, and stored at − 80 °C.

### Histopathological explorations

For histopathological explorations, kidney tissue samples were fixed in a 10% neutral buffered formalin solution. Tissue specimens were dehydrated in scaling ethanol concentration, rinsed with xylene, immobilized in paraffin wax, and split at a 5-µm thickness. The sections were fixed stained by hematoxylin and eosin on glass slides (H&E) (Bancroft and Layton 2013). The following scores were applied for the Grading System for Renal Lesions: 0, normal histology; 1, tubular epithelial cell degeneration, without significant necrosis or apoptosis; 2, tubular epithelial cell necrosis and apoptosis < 25%, tubular epithelial cell necrosis and apoptosis < 50%; 3, tubular epithelial cell necrosis and apoptosis < 75%; 4, tubular epithelial cell necrosis and apoptosis ≥ 75%; and 5, tubular epithelial cell necrosis and apoptosis ≥ 75% (Zhang et al. 2008).

### Biochemical assessments

Blood biochemical parameters urea, creatinine (Create), total-protein (TP), and albumin (ALB) were measured in serum using VITROS 350 Reference Fluid, Micro Slide Assay (Dry Chemistry system, Ortho-Clinical Diagnostics, Inc., Johnson & Johnson, Linden, NJ, USA) at the Regional Center for Food and Feed (RCFF), Agricultural Research Center, Giza, Egypt.

### Preparation of kidney homogenates

The kidney tissues’ samples were homogenized in ice-cold potassium-phosphate-buffered saline (50 mM, pH 7.4, 1/10 weight/volume to prepare a 10% (w/v)) kidney homogenates (Kadir et al. 2013), using a Universal Laboratory aid Teflon Homogenizer (type MPW-309, Poland), centrifuged using a universal cooling centrifuge (16R, Germany) at 1200 g for 15 min at 4 °C. The kidney homogenates were used to assess the oxidative stress parameters, antioxidant enzymes activity, as well as sodium and potassium level assessments.

### Determining oxidative stress parameters and antioxidant enzymes

Biodiagnostics kits (Cairo, Egypt) were used to determine the levels of malondialdehyde (MDA) (Satoh 1978), nitric oxide (NO) (Montgomery and Dymock 1961), hydrogen peroxide (H_2_O_2_) (Fossati et al. 1980), the reduced glutathione (GSH) contents (Ellman 1959), as well as superoxide dismutase (SOD) (Nishikimi et al. 1972) and catalase (CAT) (Aebi 1974) activities in the kidney homogenates, according to the mentioned procedure.

### Assessment of sodium and potassium levels

Sodium (Na^+^) and potassium (K^+^) levels were assessed in the kidney tissues using commercial kits (DiaSys Diagnostic Systems GmbH, Germany) according to the experimental procedure.

### Assessment of the trace elements: calcium and magnesium levels

The kidney tissue samples of different studied groups were digested in mixtures of concentrated nitric acid (HNO_3_) and hydrogen peroxide (H_2_O_2_) (5:1 v/v) until overall digestion of organic materials, using Milestone MLS-1200 Mega, High-Performance Microwave Digester Unit, Italy. The calcium (Ca^2+^) and magnesium (Mg^2+^) levels were estimated in the prepared tissue samples, using an atomic absorption spectrophotometer (AAS, Thermo Scientific, iCE 3000, England), at the RCFF.

### The inflammatory markers assessment by ELISA technique

ELISA kits for rats from MyBioSource were used for the assessment of interleukin-1 beta (IL-1β, Cat.No:MBS825017), interleukin-2 (IL-2, Cat.No:MBS2701041), interleukin-4 (IL-4, Cat.No:MBS2883072), interleukin-6 (IL-6, Cat.No:MBS355410), tumor necrosis factor-alpha (TNF-α, Cat.No:MBS824824), and nuclear factor-kappa B (NF-κB, Cat.No:MBS268833) levels in the kidney tissues. Analyses were performed in line with the manufacturer’s protocol for the commercial kits.

### Hypoxic induced factor-1 alpha assessment

ELISA kit for rats, from MyBioSource, was used to assess the hypoxic induced factor-1 alpha (HIF-1α, Cat.No:MBS028091) in the rats’ kidney tissues, according to the catalog’s instruction.

### Acetylcholinesterase activity assessment

Elabscience ELISA commercial kit for rats was used to assess the acetylcholinesterase activity (AchE, Cat. No: E-EL-R0355), in the rats’ kidneys tissues, according to the catalogs’ instructions.

### Angiotensin-converting enzyme activity assessment

Elabscience ELISA commercial kits for rats were used to assess the activity of angiotensin-converting enzymes ACE I (Cat. No: E-EL-R2401) and ACE II (Cat. No: E-EL-R2453), in the rats’ kidneys tissues, according to the catalogs’ instructions.

### Real-time quantitative reverse transcription–polymerase chain reaction

Total RNA in kidney tissues was extracted using Qiagen kit (USA) and quantified as formerly explained (Salem and Ismail 2021). The sequences of PCR primer pairs of inducible nitric oxide synthase (iNOS) and endothelial nitric oxide synthase (eNOS) and the housekeeping reference gene beta-actin (β-actin) with the corresponding bank gene accession number are denoted in the Supplementary data Table 1. The relative expression ratios of iNOS and eNOS genes were standardized to β-actin and calculated via the expression 2^−ΔΔCt^ (Pfaffl 2001); the experimental details are presented in the Supplementary Data 1.1.

### Western blotting analysis

TRIzol reagent was used to extract tissue proteins, which was estimated by Lowry’s method for total protein concentrations (Lowry et al. 1951). Twenty micrograms of protein per lane was isolated with 10% sodium dodecyl sulfate (SDS) polyacrylamide gel electrophoresis, then transported to polyvinylidene fluoride (PVDF) membranes. Membranes were then incubated at room temperature for 2 h with a blocking solution (5% nonfat dried milk/10 mM Tris–HCl/pH 7.5/100 mM NaCl/0.1% Tween 20). Membranes were incubated overnight at 4 °C with the designated primary antibodies (phospho-nuclear factor kappa-light-chain-enhancer of activated B cells p65 subunit (p-NF-κB p65, Ser536) monoclonal antibody (dilution factor is 1:1000, T.849.2, Cat. No: MA5-15,160), B1 bradykinin receptor rabbit polyclonal antibody (BDKRB1 (B1R), dilution factor is1:1000, Cat No: PA5-77,292, B2 bradykinin receptor rabbit polyclonal antibody (BDKRB2 (B2R), dilution factor is 1:500, Cat. No: 720288), and β-actin (dilution factor is 1:500, Cat. No: MA5-1140), Invitrogen, Thermo Fisher Scientific), then incubated with a mouse anti-rabbit secondary monoclonal antibody coupled to horseradish peroxidase (dilution factor is 1:10,000), at room temperature for 2 h. Chemiluminescence detection was achieved using the Amersham detection kit. After each incubation process, the membranes were washed several times (with 10 mM Tris–HCl/pH 7.5/100 mM NaCl/0.1% Tween 20) at room temperature. Then, the amount of the analyzed protein was computed by densitometric analysis using BioRad software for image and gel analysis, USA. The results were standardized to β-actin protein expression.

### Assessment of the micro-RNA expression

Total miRNA in kidney tissues was isolated using mirVana™ miRNA isolation kit (Life Technologies Corporation, USA (Cat. No:AM1560), reverse transcribed to cDNA using TaqMan™ microRNA reverse transcription kit (Thermo Fisher Scientific, USA, Cat. No:4366597) applying the company’s information and quantified using the rno-miRNA-129–1-3pmiRNA PCR kit (Cat. No: MBS8297717, NC-051339.1), rno-miR-200c-3p miRNA PCR kit (Cat. No: MBS8297433, NC_051339.1), and rno-miR-210-3p PCR kit (Cat. No: MBS8297441, NC_051339.1) from MyBioSource. These kits contain SYPR green dye. The relative expression levels of each miRNA were calculated as 2^−ΔΔCt^ (Pfaffl 2001), after normalization to the expression of the housekeeping gene RNU6 in each examined sample.

### Statistical analysis

The Statistical Package for Social Science Software program (SPSS, version 21.0) and Microsoft Excel were used to analyze the data. The data were explained as mean ± standard error (SE). ANOVA (one-way analysis of variance) with Tukey post hoc multiple comparisons was used to test the variation in the means of the variables among groups. The probability of *P* < 0.05 was thought to be significant.

## Results

### Histopathology

**Fig. 1:**
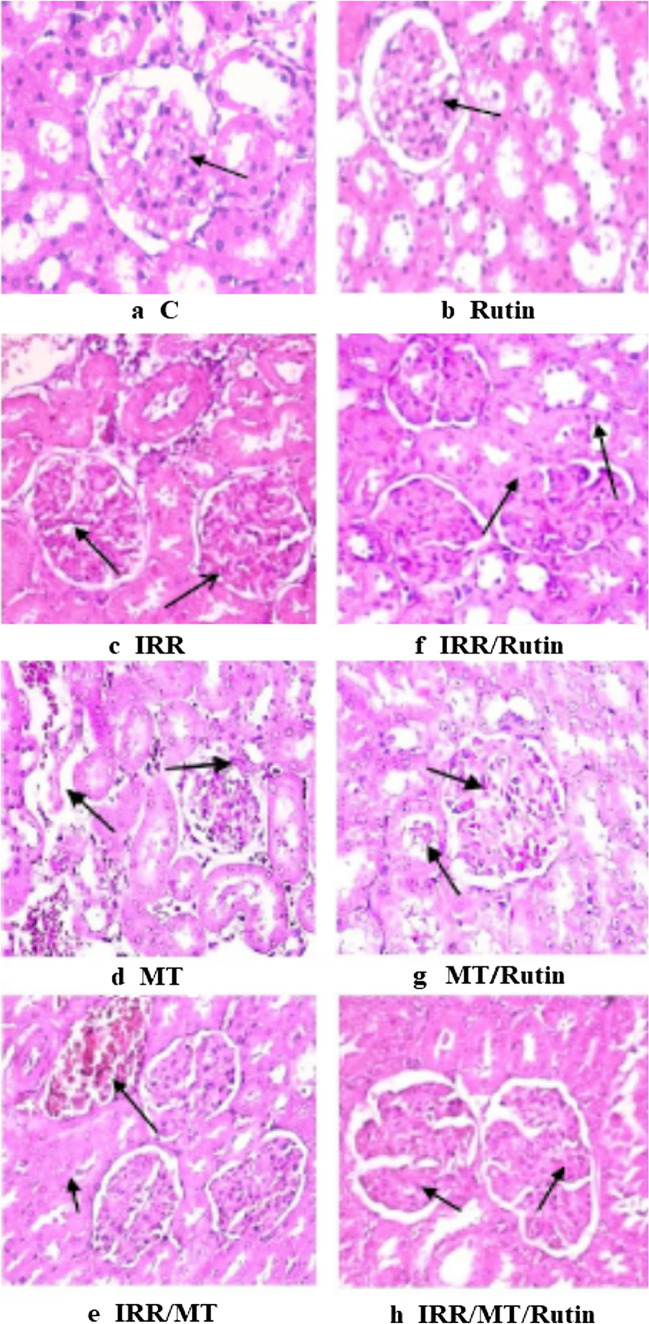
Histopathological changes in the kidney tissues. Photomicrograph of kidney tissue sections showing (**a**: C, control (G1); and **b**: Rutin (G2), rutin treated animals) normal structure glomeruli and renal tubules arrow (**c**: IRR (G3), gamma-irradiated animals); shrinkage of capillary tufts, interstitial edema, and few mononuclear cell infiltration arrow (**d**: MT (G4), malathion-treated animals); swelling of tubular epithelial lining and congestion of glomerular tufts arrow (**e**: IRR/MT (G5), gamma-irradiation/malathion-treated animals); severe congestion and dilatation of capillary tufts arrow (**f**: IRR/Rutin (G6), gamma-irradiation/rutin-treated animals); swelling and granularity of tubular epithelial lining and congestion of interstitial capillaries arrow (**g**: MT/Rutin (G7), malathion/rutin-treated animals); and intact and regular arrangement of tubular epithelial lining arrow (**h**: IRR/MT/Rutin (G8), gamma-irradiation/malathion/rutin-treated animals) mild swelling of tubular epithelial lining and hypercellular of capillary tufts arrow (H&E × 400)

The control (C, G1) and rutin-treated animal (Rutin, G2) kidney tissue sectors demonstrated normal histological structure designated by circumscribing glomeruli with the ordinary construction of capillary tufts and Bowman’s capsule. The renal tubules of both proximal and distal convoluted tubules displayed intact epithelial lining and regular arrangement (score 0) (Fig. 1 a and 1 b).

The kidney tissue section from the animal’s group exposed to 3 Gy as a single dose of γ-radiation (IRR, G4) exhibited minor histopathological modifications of renal tubular epithelial lining performed in swelling of cell lining appearance. Tubular epithelial cells displayed degeneration, with insignificant necrosis or apoptosis. The glomeruli demonstrated severe congestion of glomerular tufts (score 1) (Fig. 1 c). The kidney tissues of MT (G4) showed shrinkage of capillary tufts with the widening of Bowman’s space of some glomeruli. The renal tubules displayed epithelial cell degeneration with marked swelling of tubular epithelial lining associated with narrowing and occlusion of tubular lumen by albuminous and cellular casts. Tubular epithelial cell necrosis and apoptosis < 25% (score 2) were seen. Interstitial edema characterized by widening spaces in-between the renal tubules and few mononuclear cell infiltration mainly lymphocytes and macrophages were noticed (Fig. 1 d).

On the other side, kidney tissue sections of the animals intoxicated with IRR/MT (G5) showed hypercellularity of capillary tufts with narrowing the Bowman’s space of some glomeruli. Degeneration of renal tubular epithelial lining appeared in the form of swelling and granularity of its cytoplasm. Intratubular albuminous eosinophilic casts and congestion of interstitial capillaries were seen. Tubular epithelial cell necrosis and apoptosis < 50% were seen (score 3) (Fig. 1 e).

The kidney tissue sections of the irradiated animals treated with rutin (IRR/Rutin, G6) showed a normal histological structure of glomerular capillary tufts and Bowman’s capsule. The renal tubules of both proximal and distal convoluted tubules showed intact epithelial lining and regular arrangement (score 0) (Fig. 1 f). On the other hand, kidney tissues of animals co-treated with MT and rutin (MT/Rutin G7) showed mild histological changes of renal tubular epithelial lining appearing in the form of swelling of cell lining. Tubular epithelial cell degeneration, without significant necrosis or apoptosis, was observed. The glomeruli showed a mild degree of glomerular tufts congestion score (1) (Fig. 1 g). In the same direction, the kidney tissue sections of the irradiated animals co-treated with malathion and rutin (IRR/MT/Rutin, G8) showed mild histological changes of renal tubular epithelial lining appeared in the form of mild swelling of cell lining. Tubular epithelial cell degeneration was seen, without significant necrosis or apoptosis. The glomeruli showed a hypercellular capillary tufts score (1) (Fig. 1 h).

### Biochemical analysis

**Fig. 2:**
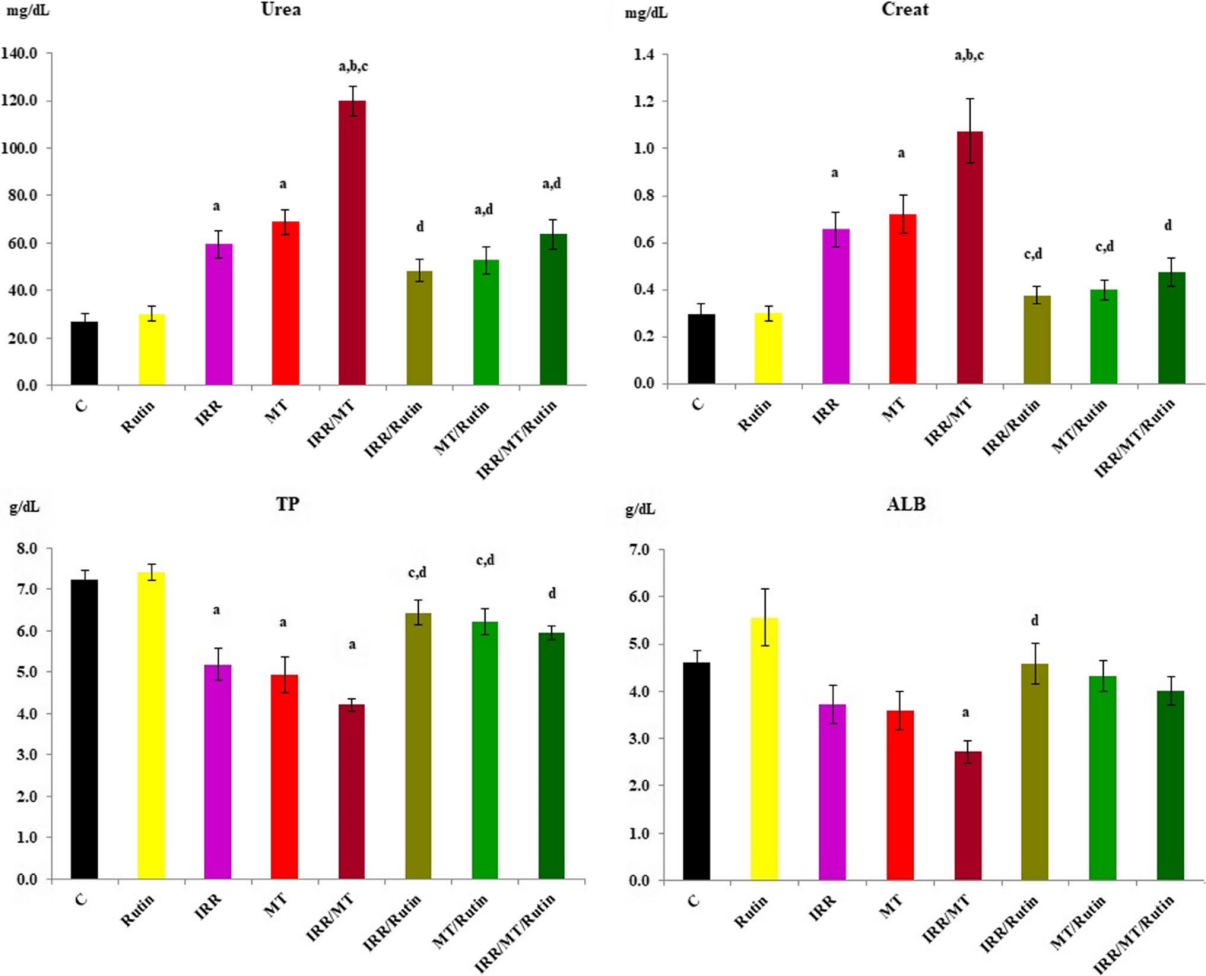
Biochemical parameters in the serum. C, control; rutin, rutin-treated animals; IRR, gamma-irradiated animals; MT, malathion-treated animals; IRR/MT, gamma-irradiation/malathion-treated animals; IRR/rutin, gamma-irradiation/rutin-treated animals; MT/rutin, malathion/rutin-treated animals; IRR/MT/rutin, gamma-irradiation/malathion/rutin-treated animals; Creat, creatinine; TP, total protein; ALB, albumin. The statistical significance to control, IRR, MT, and IRR/MT are denoted by a, b, c, and d, respectively, at *p* < 0.05. Statistical significance was analyzed by one-way ANOVA with Tukey post hoc multiple comparisons

The data demonstrated that IRR exposure, MT administration, and their combined toxic effect (IRR/MT) verified substantial increases (*P* < 0.01) in urea and creatinine levels, but significant declines (*P* < 0.01) in TP and ALB levels occurred in the blood serum, as compared to the controls. However, rutin treatment recovered the kidney function via regulation of urea, creatinine, TP, and ALB levels in the serum of IRR, MT, and IRR/MT intoxicated animals toward their corresponding control values (Fig. 2).

### Oxidative stress and antioxidants in the kidney

**Fig. 3:**
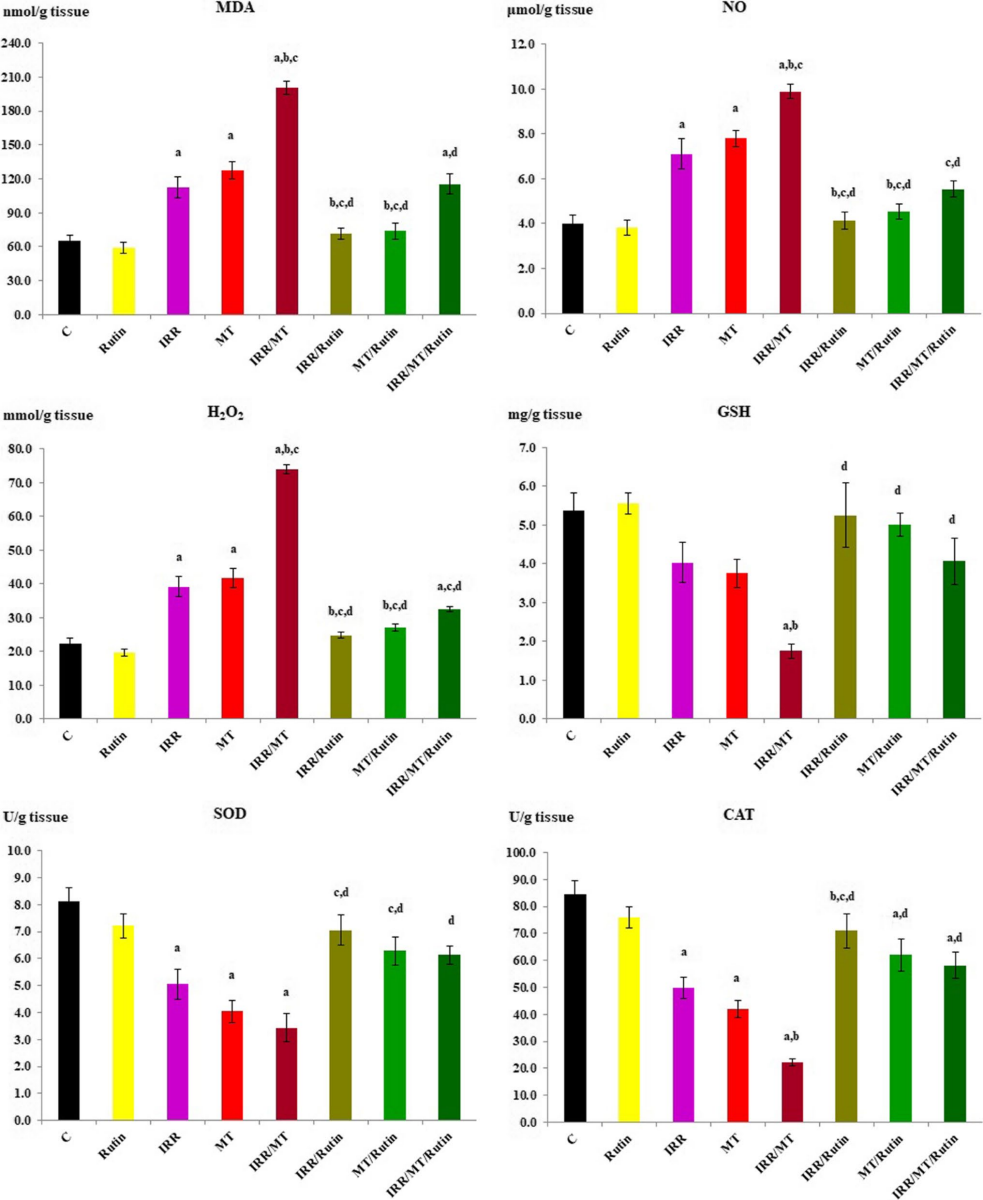
The renal antioxidant status. C, control; rutin, rutin-treated animals; IRR, gamma-irradiated animals; MT, malathion-treated animals; IRR/MT, gamma-irradiation/malathion-treated animals; IRR/rutin, gamma-irradiation/rutin-treated animals; MT/rutin, malathion/rutin-treated animals; IRR/MT/rutin, gamma-irradiation/malathion/rutin-treated animals; MDA, malondialdehyde; NO, nitric oxide; H_2_O_2_, hydrogen peroxide; GSH, reduced glutathione; SOD, superoxide dismutase; CAT, catalase. The statistical significance to control, IRR, MT, and IRR/MT are denoted by a, b, c, and d, respectively, at *p* < 0.05. Statistical significance was analyzed by one-way ANOVA with Tukey post hoc multiple comparisons

The oxidative stress and antioxidant enzymes were determined in the kidney tissues of different experimental groups. The levels of MDA, NO, and H_2_O_2_ were significantly increased, accompanied by significant decreases in the GSH contents and suppression of the SOD and CAT activities in the kidney tissues of IRR, MT, and IRR/MT groups, as compared to the control values. However, rutin treatment exhibited depression in the levels of MDA, NO and H_2_O_2_, as well as elevation of the GSH contents and enhanced the activity of the antioxidant enzymes (SOD and CAT) in the kidney tissues of IRR, MT, and IRR/MT intoxicated animals (Fig. 3).

### The levels of sodium and potassium

**Fig. 4:**
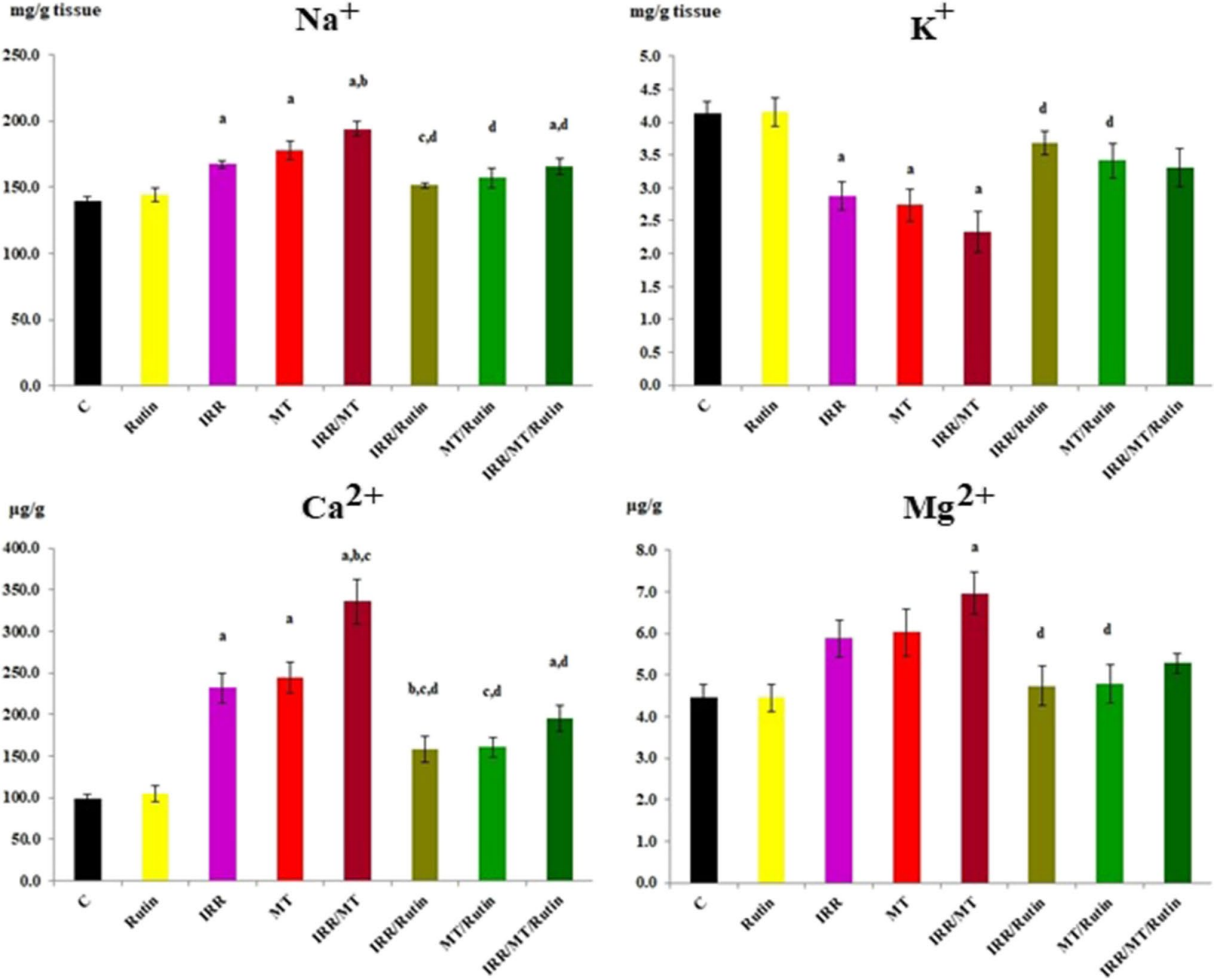
The levels of sodium, potassium, magnesium, and calcium in the kidney tissues. C, control; rutin, rutin-treated animals; IRR, gamma-irradiated animals; MT, malathion-treated animals; IRR/MT, gamma-irradiation/malathion-treated animals; IRR/rutin, gamma-irradiation/rutin-treated animals; MT/rutin, malathion/rutin-treated animals; IRR/MT/rutin, gamma-irradiation/malathion/rutin-treated animals; Na^+^, sodium; K^+^, potassium; Ca^2+^, calcium; Mg^2+^, magnesium. The statistical significance to control, IRR, MT, and IRR/MT are denoted by a, b, c, and d, respectively, at *p* < 0.05. Statistical significance was analyzed by one-way ANOVA with Tukey post hoc multiple comparisons. Sodium and potassium assessed by commercial kits. Calcium and magnesium assessed by atomic absorption technique

The level of Na^+^ was dramatically enhanced, while K^+^ level diminished, respectively, in the kidney tissues’ homogenates of the IRR, MT, and IRR/MT intoxicated animals, as compared to the control values. However, rutin treatment recovered the levels of Na^+^ and K^+^ in the kidney tissues of IRR, MT, and IRR/MT intoxicated animals (Fig. 4).

### The levels of the trace elements: calcium and magnesium

The levels of Ca^2+^ and Mg^2+^ were significantly elevated in the kidney tissues of the IRR, MT, and IRR/MT intoxicated animals, in contrast to the control values. However, rutin administration ameliorated the levels of Ca^2+^ and Mg^2+^ in the kidney tissues of IRR, MT, and IRR/MT intoxicated groups (Fig. 4).

### Inflammatory markers

**Fig. 5:**
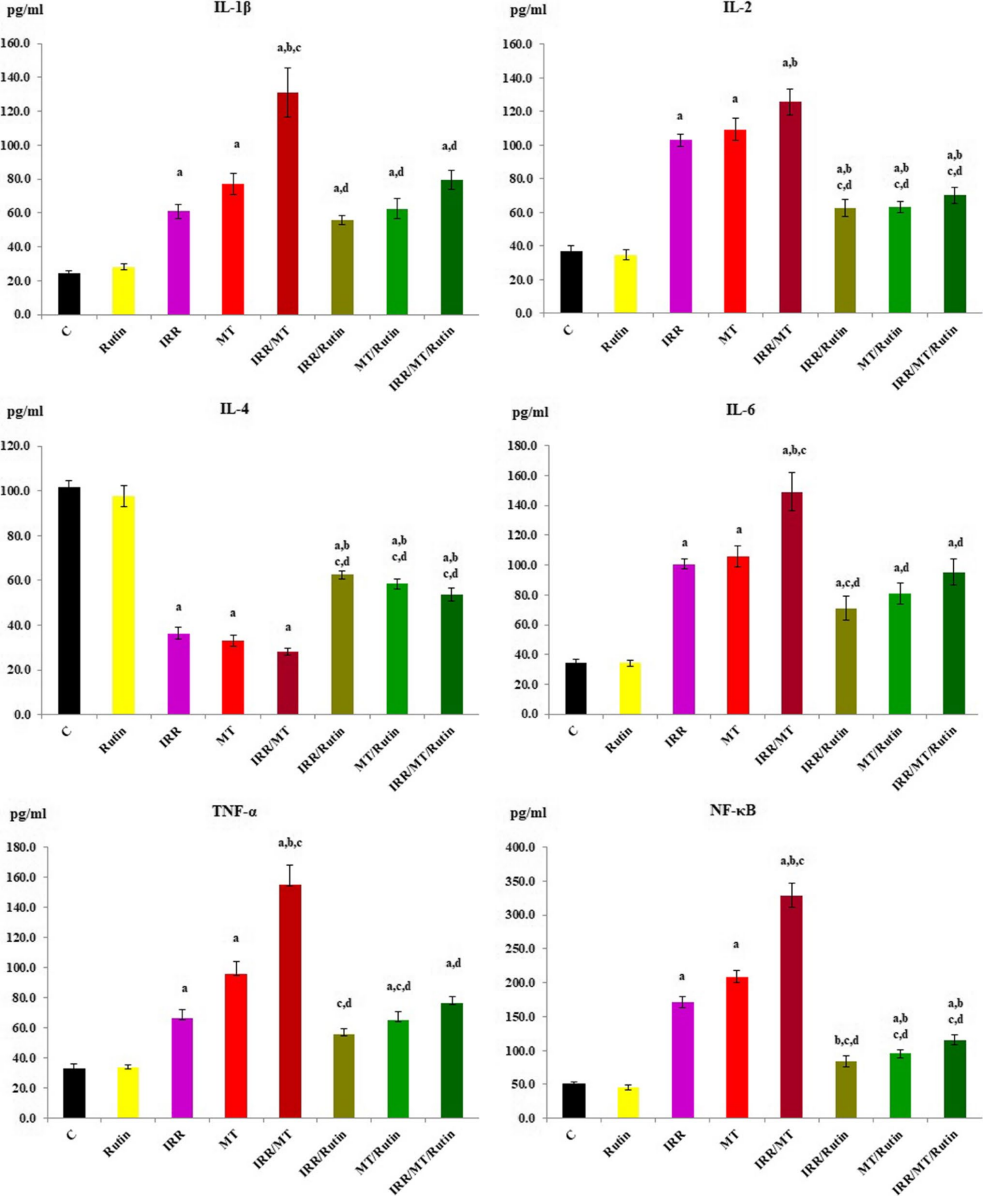
Inflammatory markers levels in the kidney tissues. C, control; rutin, rutin-treated animals; IRR, gamma-irradiated animals; MT, malathion-treated animals; IRR/MT, gamma-irradiation/malathion-treated animals; IRR/rutin, gamma-irradiation/rutin-treated animals; MT/rutin, malathion/rutin-treated animals; IRR/MT/rutin, gamma-irradiation/malathion/rutin-treated animals; IL-1β, interleukin-1 beta; IL-2, interleukin-2; IL-4, interleukin-4; IL-6, interleukin 6; TNF-α, tumor necrosis factor-alpha; NF-κB, nuclear factor–kappa B. The statistical significance to control, IRR, MT, and IRR/MT are denoted by a, b, c, and d, respectively, at *p* < 0.05. Statistical significance was analyzed by one-way ANOVA with Tukey post hoc multiple comparisons. These inflammatory markers are assessed by ELISA technique

On the other hand, the levels of the pro-inflammatory markers IL-1β, IL-2, IL-6, TNF-α, and NF-κB were elevated, while the level of IL-4 was declined in the kidney tissues of IRR, MT, and IRR/MT treated rats, as compared to the control values. However, rutin treatment regulated the inflammatory status. The levels of the pro-inflammatory markers IL-1 β, IL-2, IL-6, TNF-α, and NF-κB were reduced, while the level of IL-4 was increased in the kidney tissues of IRR/rutin, MT/rutin, and IRR/MT/rutin groups (Fig. 5).

### Hypoxia-induced factor-1α level

**Fig. 6:**
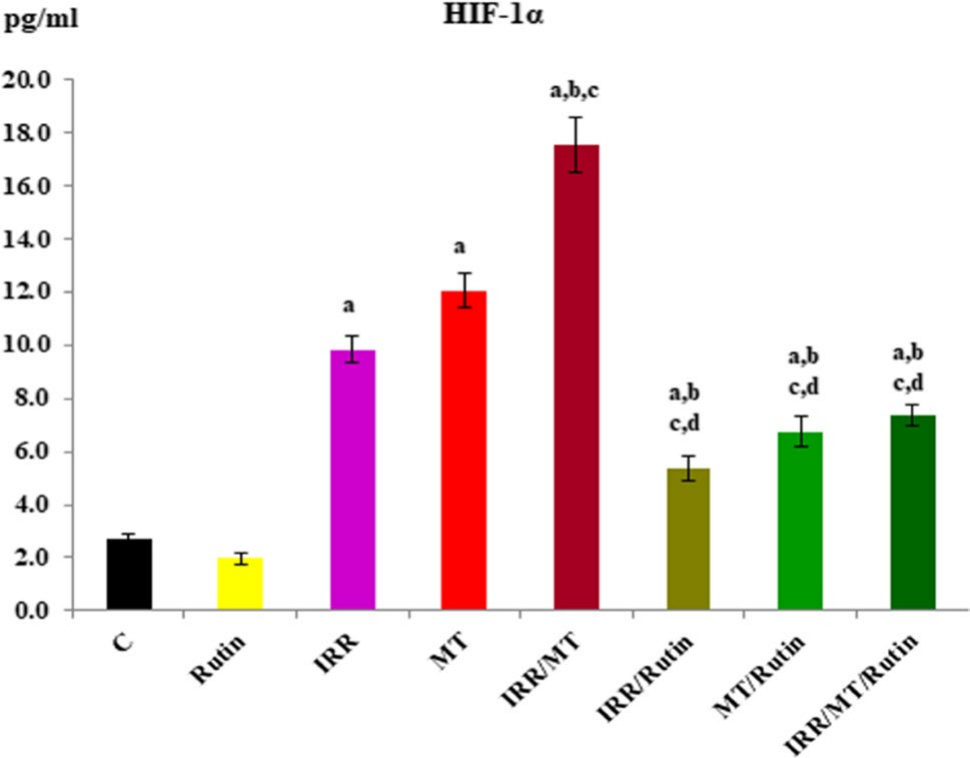
The hypoxia induced factor-1 alpha level in the kidney tissues. C, control; rutin, rutin-treated animals; IRR, gamma-irradiated animals; MT, malathion-treated animals; IRR/MT, gamma-irradiation/malathion-treated animals; IRR/rutin, gamma-irradiation/rutin-treated animals; MT/rutin, malathion/rutin-treated animals; IRR/MT/rutin, gamma-irradiation/malathion/rutin-treated animals. The hypoxia induced factor-1 alpha (HIF-1α) level is assessed by ELISA technique. The statistical significance to control, IRR, MT, and IRR/MT are denoted by a, b, c, and d, respectively, at *p* < 0.05. Statistical significance was analyzed by one-way ANOVA with Tukey post hoc multiple comparisons. HIF-1α is assessed by ELISA technique

The level of HIF-1α was significantly increased in the kidney tissues of IRR-, MT-, and IRR/MT-intoxicated animals, as compared to the control value. However, rutin treatment depressed these levels in the kidney tissues of IRR, MT, and IRR/MT intoxicated animals (Fig. 6).

### Acetylcholinesterase activity

**Fig. 7:**
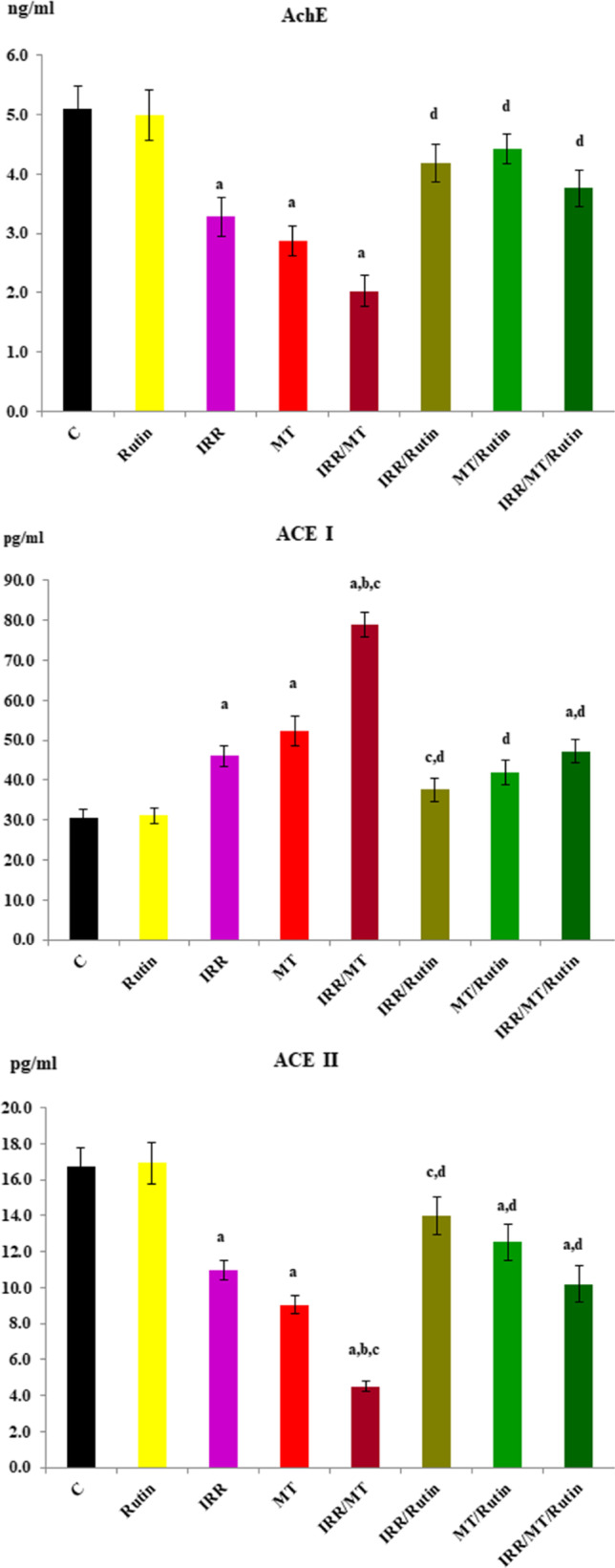
The activity of acetylcholinesterase, angiotensin I converting enzyme, and angiotensin II converting enzyme in the kidney tissues. C, control; rutin, rutin-treated animals; IRR, gamma-irradiated animals; MT, malathion-treated animals; IRR/MT, gamma-irradiation/malathion-treated animals; IRR/rutin, gamma-irradiation/rutin-treated animals; MT/rutin, malathion/rutin-treated animals; IRR/MT/rutin, gamma-irradiation/malathion/rutin-treated animals; AchE, acetylcholinesterase; ACE I, angiotensin I converting enzyme. The statistical significance to control, IRR, MT, and IRR/MT are denoted by a, b, c, and d, respectively, at *p* < 0.05. Statistical significance was analyzed by one-way ANOVA with Tukey post hoc multiple comparisons. These enzymes are assessed by ELISA technique

The activity of AchE was suppressed in the kidney tissues of IRR-, MT-, and IRR/MT-intoxicated rats, as compared to the control animals. However, rutin administration encouraged the activity of AchE in the kidney tissues of the IRR-, MT-, and IRR/MT-intoxicated animals (Fig. 7).

### Angiotensin converting enzyme activity

The activity of ACE I was recovered, while the ACE II activity was repressed in the kidney tissues of IRR-, MT-, and IRR/MT-intoxicated rats, as compared to the control group. However, rutin administration ameliorated the activities of ACE I and ACE II in the kidney tissues of the IRR-, MT-, and IRR/MT-intoxicated animals (Fig. 7).

### The gene expression ratio of inducible nitric oxide synthase (iNOS) and endothelial nitric oxide synthase (eNOS)

**Fig. 8:**
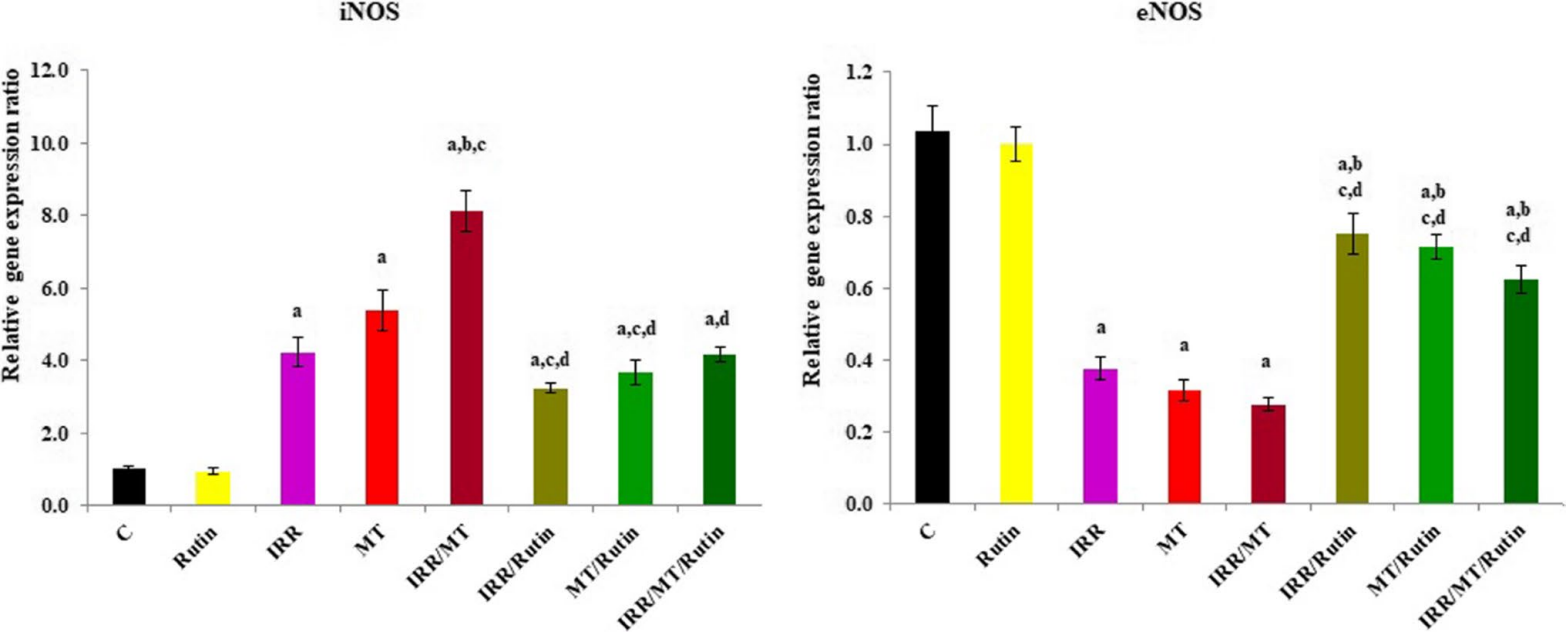
The mRNA gene expression ratio of inducible nitric oxide synthase, and endothelial nitric oxide synthase in the kidney tissues. C, control; rutin, rutin-treated animals; IRR, gamma-irradiated animals; MT, malathion-treated animals; IRR/MT, gamma-irradiation/malathion-treated animals; IRR/rutin, gamma-irradiation/rutin-treated animals; MT/rutin, malathion/rutin-treated animals; IRR/MT/rutin, gamma-irradiation/malathion/rutin-treated animals; iNOS, inducible nitric oxide synthase; eNOS, endothelial inducible nitric oxide synthase. The statistical significance to control, IRR, MT, and IRR/MT are denoted by a, b, c, and d, respectively, at *p* < 0.05. Statistical significance was analyzed by one-way ANOVA with Tukey post hoc multiple comparisons. The levels of iNOS and eNOS are assessed by RT-PCR technique

The mRNA gene expression ratios of iNOS were up-regulated, while the mRNA gene expression ratios of eNOS were down-regulated, respectively, in the kidney tissues of IRR-, MT-, and IRR/MT-intoxicated rats, compared to the control animals. However, rutin treatment regulated the mRNA gene expression ratios of iNOS and eNOS in the kidney tissues of the IRR-, MT-, and IRR/MT-intoxicated groups (Fig. 8).

### Western blot analysis of p-NF-κB p65

**Fig. 9:**
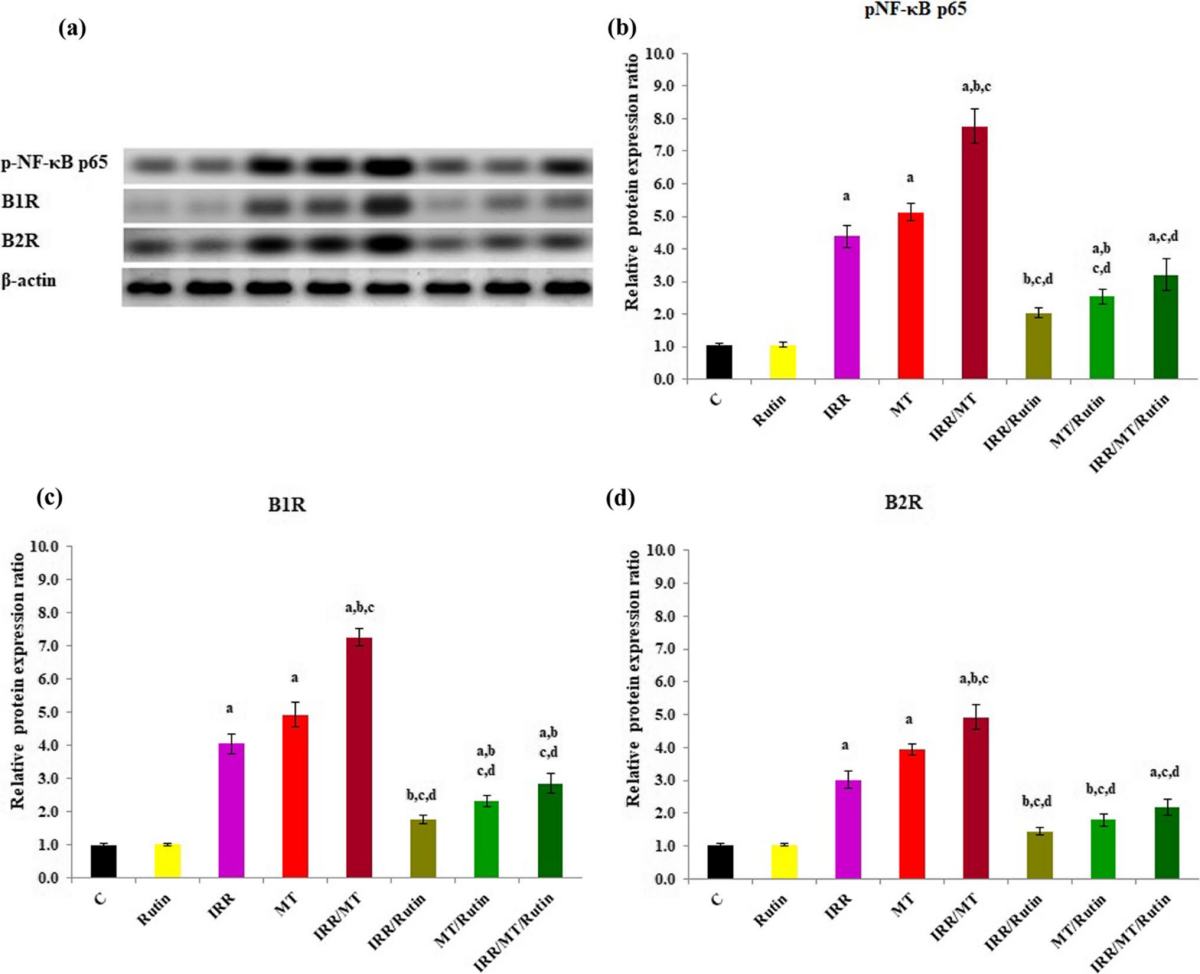
Western blots analysis (**a**) and the expression ratio of phospho-nuclear factor–kappa B p65 (**b**), B1 bradykinin receptor (**c**) and B2 bradykinin receptor (**d**) relative to β-actin in the rat kidney tissues of different experimental groups. C, control; rutin, rutin-treated animals; IRR, gamma-irradiated animals; MT, malathion-treated animals; IRR/MT, gamma-irradiation/malathion-treated animals; IRR/rutin, gamma-irradiation/rutin-treated animals; MT/rutin, malathion/rutin-treated animals; IRR/MT/rutin, gamma-irradiation/malathion/rutin-treated animals; p-NF-κB p65, phospho-nuclear factor kappa-light-chain-enhancer of activated B cells p65 subunit; B1R, B1 bradykinin receptor rabbit polyclonal antibody (BDKRB1); B2R, B1 bradykinin receptor rabbit polyclonal antibody (BDKRB2). The statistical significance to control, IRR, MT, and IRR/MT are denoted by a, b, c, and d, respectively, at *p* < 0.05. Statistical significance was analyzed by one-way ANOVA with Tukey post hoc multiple comparisons. The same amount of protein of the kidney tissues was analyzed by Western blots. Rutin suppressed the up-regulation of p-NF-κB p65, B1R, and B2R protein expressions triggered by gamma-irradiation (IRR) and malathion (MT)

The protein expression ratios of p-NF-κB p65 were up-regulated in the kidney tissues of the IRR-, MT-, and IRR/MT-intoxicated animals. However, rutin treatment down-regulated the p-NF-κB p65 protein expression ratios in the kidney tissues of IRR-, MT-, and IRR/MT-intoxicated animals (Fig. 9).

### Western blot analysis of bradykinin receptors

The protein expression ratios of bradykinin receptors B1R and B2R were up-regulated in the kidney tissues of IRR-, MT-, and IRR/MT-intoxicated rats, as compared to the control ratios. On the contrary, rutin treatment down-regulated the relative protein expression ratios ofB1R and B2R in the kidney tissues of the IRR-, MT-, and IRR/MT-intoxicated groups (Fig. 9).

### Micro-RNA gene expression

**Fig. 10:**
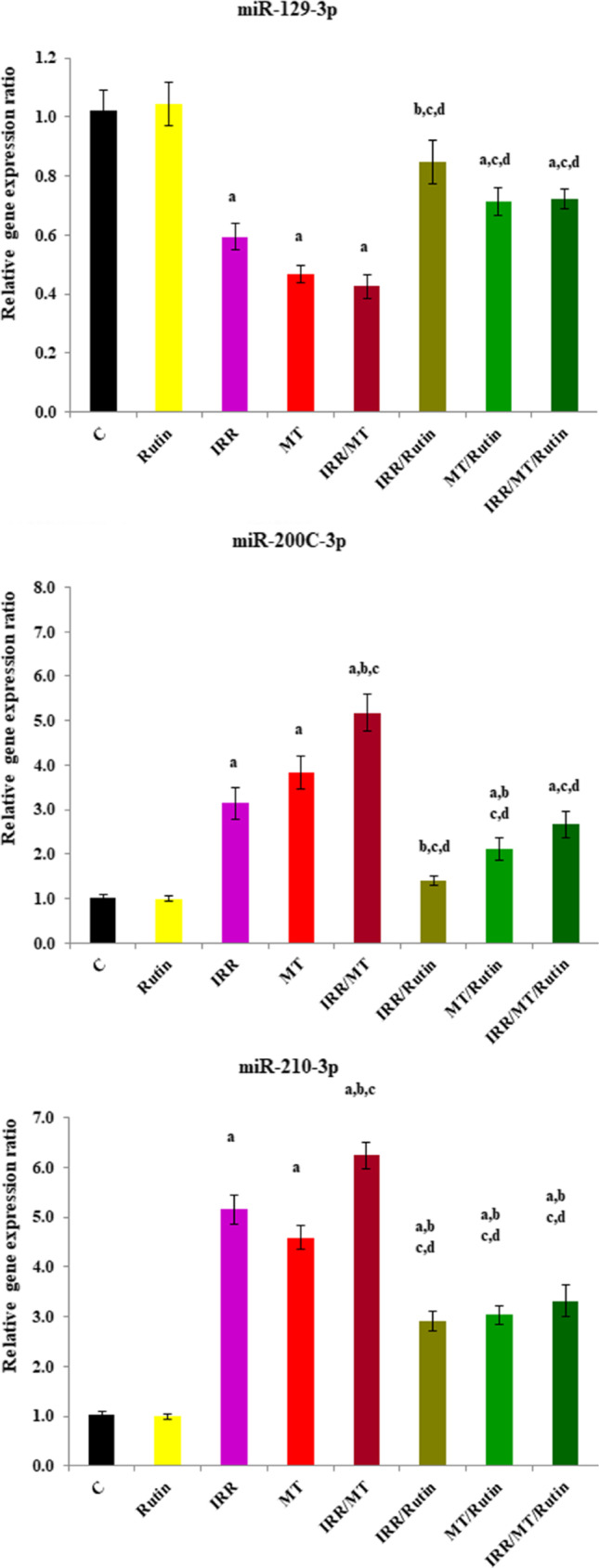
Relative gene expression ratio of micro-RNA 129-3p, micro-RNA 200c-3p, and micro-RNA 210-3p in the kidney tissues. C, control; rutin, rutin-treated animals; IRR, gamma-irradiated animals; MT, malathion-treated animals; IRR/MT, gamma-irradiation/malathion-treated animals; IRR/rutin, gamma-irradiation/rutin-treated animals; MT/rutin, malathion/rutin-treated animals; IRR/MT/rutin, gamma-irradiation/malathion/rutin-treated animals. The statistical significance to control, IRR, MT and IRR/MT are denoted by a, b, c, and d, respectively, at *p* < 0.05. Statistical significance was analyzed by one-way ANOVA with Tukey post hoc multiple comparisons

The mRNA gene expression ratios of miR-129–1-3p were down-regulated; however, the mRNA gene expression ratios of miR-200c-3p and miR-210-3p were up-regulated in the kidney tissues of the IRR-, MT-, and IRR/MT-intoxicated rats, as compared to the control ratios. However, rutin treatment up-regulated the mRNA gene expression ratios of miR-129–1-3p and down-regulated the relative gene expression ratios of miR-200c-3p and miR-210-3p in the kidney tissues of the IRR-, MT-, and IRR/MT-intoxicated groups (Fig. 10).

## Discussion

Malathion belongs to the largest and most diversified group of pesticides, organophosphate insecticides. It is widely used in public health and agricultural fields. Consequently, it demonstrates harmful environmental effects, which results in severe acute and chronic ailments to different organs, including the kidneys (Baiomy et al. 2015; Ince et al. 2017). Several studies have demonstrated that MT administration is associated with renal histological modifications inducing renal damage (Selmi et al. 2018). In this investigation, rats exposed to a single dose of IRR demonstrated physiological variations in the kidney functions concomitant with mild histopathological changes. It is documented that gamma-irradiation exposure boosted the alterations in the renal functions (Elkady and Ibrahim 2016; Ismail et al. 2016a; Salem and Ismail 2021). However, chronic toxicity of MT established histopathological alterations in the kidney tissues with marked tubular epithelial cell necrosis and apoptosis, associated with significant increases in kidney functions and urea and creatinine levels, accompanied by significant decreases in TP and ALB levels in the serum of MT-treated animals. These results are confirmed by the early study of Baiomy and co-workers (Baiomy et al. 2015). The combined treatment IRR/MT augmented these changes, whereas exposure to gamma irradiation prior to MT administration enhanced the acquired oxidative stress, thus amplifying the alterations in kidney functions and kidney architecture. Likewise, enhancement of the levels of Na^+^ and the trace elements Mg^2+^ and Ca^2+^ associated with declines in the and K^+^ levels were detected in the kidney tissues of MT- and IRR-intoxicated rats, which was magnified in IRR/MT-intoxicated rats. IRR exposure and/or MT chronic administration stimulates kidney dysfunction and elicits electrolyte imbalance. The oxidative stress developed in the kidney tissues assists membrane permeability alterations, tubular epithelium, and tubulointerstitial components destruction, amino acid oxidative-deamination, and protein catabolism leading to renal impairment (Baiomy et al. 2015; Ismail et al. 2016a; Salem and Ismail 2021). The amino acid metabolism is altered in the liver of γ-irradiated rats (Zaher et al. 2016) or MT-intoxicated animals (Khalifa and Alkhalaf 2020); thus, TP and ALB levels were diminished in the blood circulation. However, rutin treatment protected the intoxicated kidney due to counteracting the kidney microstructure changes (Diwan et al. 2017).

In the current investigation, MT demonstrates alterations in the kidney functions accompanied by renal oxidative stress. MT suppressed the activity of antioxidant enzymes such as SOD and CAT, thus encouraging MDA accumulation in the kidney tissues (Selmi et al. 2018; Akbel et al. 2018; Salari et al. 2021). The data of this investigation demonstrates renal impairment accompanied by elevated levels of MDA and NO, as well as depression of the CAT and SOD activities and depressed GSH contents in the MT- and IRR-intoxicated animals, which is augmented in IRR/MT group. Oxidative stress has been linked to MT, similar to other organophosphate insecticides (Dhouib et al. 2015). An imbalance between the systemic expression of ROS and a biological system’s ability to quickly detoxify the reactive intermediates is referred to as oxidative stress. The release of ROS by the mitochondrial respiratory chain is linked to oxidative stress, leading to a significant decay in the antioxidant defense effectiveness, thus, initiating lipid peroxidation and enzyme suppression in addition to destruction of DNA and most important biomolecules. The initial defense line against free radical-induced oxidative stress is the antioxidant enzymes SOD, CAT, and glutathione peroxidase (GPx). Superoxide radicals are known to be converted by SOD to H_2_O_2_ and molecular oxygen. CAT is responsible for the enzymatic breakdown of H_2_O_2_ to molecular oxygen and water. GPx catalyzes the interaction between glutathione and H_2_O_2_. MT exposure has been shown to induce lipid peroxidation in rodent tissues. It has been proposed that superoxide radicals, either alone or after being converted to H_2_O_2_, promote oxidation of the cysteine in the enzyme, lowering SOD activity. As a result, suppressed SOD activity could indicate cellular oxidative stress because of MT exposure. Accordingly, MT can instigate an oxidative imbalance by reducing the intracellular level of GSH and can also suppress the activities of GSH-dependent enzymes, GST and GPx, resulting in oxidative processes and increased cell death (Dhouib et al. 2015). In this investigation, the levels of MDA and H_2_O_2_ were significantly increased, accompanied by significant decreases in the GSH contents and suppression of the SOD and CAT activities in the kidney tissues of IRR-, MT-, and IRR/MT-intoxicated animals. Similar outputs were documented previously (Dhouib et al. 2015; Ince et al. 2017). Under oxidative stress circumstances, oxygen and energy supply is depleted; accordingly, breakdown of the mitochondrial oxidative phosphorylation is constructed, and the cells undergo hypoxia, leading to enhancement of the HIF-1α levels in the cells (Schödel et al. 2009; Salem and Ismail 2021), which explains the elevated levels of HIF-1α in the kidney tissues of IRR-, MT-, and IRR/MT-intoxicated animals in the current investigation. However, rutin administration reversed these toxic symptoms due to its potent antioxidant and free radical scavenging activities. Several natural products containing polyphenolic and flavonoid moieties were used as radioprotective agents as well as neutralizing agents of MT toxicity (Diwan et al. 2017; Karabag-Coban et al. 2016; Ahmed et al. 2009; Alsherbiny et al. 2019; Khalifa and Alkhalaf 2020; Salari et al. 2021).

On the other hand, nitric oxide synthases (NOS) are a group of enzymes that catalyze the conversion of L-arginine to NO. NO is a crucial chemical in cellular signaling. NO has multiple functional roles in the kidneys, including renal blood flow, tubular regulation, and glomerular filtration, all of which affect overall body homeostasis. NO overproduction is involved in severe kidney dysfunctions and the pathophysiology of various diseases affecting kidney functions. The intra-renal NOS isoforms are relevant to NO-mediating kidney functioning (Mungrue et al. 2003), and the regulation of the NOS expressions has an effective role in NO activity in kidney tissues (Holmqvist et al. 2005). iNOS and eNOS are involved in NO production, whereas NO stimulated by iNOS evokes tissue destruction. However, NO emitted by eNOS develops tissues protection (Betz et al. 2012). This released NO is involved in mediating organ toxicity by inflammatory response during oxidative stress conditions (Ismail et al. 2016b). The gene expression of iNOS was up-regulated, while that of eNOS was down-regulated in parallel with elevated levels of NO in the current investigation. Exposure to gamma-irradiation creates ROS and RNS (reactive nitrogen species), which prompts an inflammatory response and encourages the release of several pro-inflammatory cytokines such as TNF- and IL-6. The translocation of NF-κB from the cytoplasm to the nucleus could be stimulated by activated TNF-α and induction of the NF-κB signaling pathway, which includes the activation of different kinases, leading to phosphorylation and degradation of IκB, the inhibitor of NF-κB. The up-regulation of NF-κB levels activates the iNOS and the pro-inflammatory cytokines, such as TNF-α and IL-6 in an endless loop. The released IL-6 stimulates the production of iNOS and COX-2, which leads to an increase in prostaglandin PGE2 generation and increased oxidative stress in the intoxicated tissues (Zaher et al. 2016). The chronic inflammation-nephropathy observed due to MT toxicity is correlated with the alterations in the kidney architecture (Jensen and Whatling 2010). Moreover, these inflammatory markers trigger the assembly of ROS and thus worsen kidney performance (Sureshbabu et al. 2015). Renal inflammation possesses a critical role during the progress of CKD, which enhances the occurrence of cardiovascular diseases and other metabolic diseases (Gu et al. 2020). The data demonstrates an elevated levels of IL-1β, IL-6, and TNF-α, and subsequently enhanced NF-κB level in the kidney tissues due to the exposure of animals to IRR or administration of MT. However, the exposure to IRR prior to MT chronic administration augments this inflammatory response in the kidney tissues of IRR/MT animal group. On the other hand, the IL-2 level is enhanced in the kidney tissues of IRR-, MT-, and IRR/MT-intoxicated animals. Hefnawy et al. (2016) reported that IL-2 level was elevated in the serum of MT-intoxicated patients, which could be related to the activation of the lymphocytes, T-cell proliferation, and enhancement of the immune response (Seth et al. 2008). On the contrary, rutin administration to IRR-, MT-, and IRR/MT-intoxicated animals exhibits anti-inflammatory effects, which could be related to the enhancement of the level of anti-inflammatory cytokine IL-4 in the kidney tissues. IL-4 level was declined in the kidney tissues of intoxicated groups due to IRR exposure and MT administration in the current investigation. Improvement of IL-4 can suppress the release of the pro-inflammatory cytokines IL-1β, IL-6, and TNF-α and can recover other anti-inflammatory mediators (Gu et al. 2020), accordingly regulating the NF-κB and its inflammatory pathways.

Inhibition of AchE is one of the most critical drawbacks of MT exposure due to encouragement of the cholinergic route (Bartling et al. 2007; Gupta et al.. 2019). AchE activity is depressed in the serum and tissues due to MT toxicity (Akhgari et al. 2003; Ince et al. 2017). AchE hydrolyzes acetylcholine to choline; accordingly, the inhibition of AchE encourages the accumulation of acetylcholine in kidney tissues. Acetylcholine enhances the renal blood flow and urinary nitrate/nitrite excretion in kidney tissues and encourages water and electrolyte clearances. However, in chronic renal diseases, this process is diminished. Moreover, in chronic renal failure, acetylcholine prompts alterations in these components, obstructs tubular sodium reabsorption, and diminishes water excretion. Thus, the accumulation of acetylcholine can suppress water and electrolyte clearances in kidney tissues and can trigger alterations in the glomerular filtration rate (Vander 1964). MT is designated as an AchE inhibitor, whereas the phosphate group of MT is covalently attached to the serine residue at the active site of the enzyme (Colovic et al. 2013; Gupta et al. 2019). However, rutin administration re-activates AchE activity in the kidney tissues of IRR-, MT-, and IRR/MT-intoxicated animals and exhibited regulatory effects on AchE. In a previous investigation, rutin obstructed the AchE activity (Adefegha et al. 2018), whereas it developed a structural modification of AchE after their connection at Trp 279 or Trp 84 tryptophan moiety, forming rutin-AchE complex (Yan et al. 2018). On the other hand, in the current investigation, rutin administration recovers AchE activity that could be attributed to its potent antioxidant and scavenger activity of ROS. The mechanism of AchE reactivation by rutin warranted further investigations.

It is well known that MT exposure triggers kidney dysfunctions, characterized by alterations of urea, creatinine, TP, and ALB levels and structural modifications in the kidney tissues (Badr 2020). On the other hand, organophosphates are implicated in CKD development (Wan et al. 2021). Hypertension is documented as one of the risk features of CKD (Levey and Coresh 2012). The kidneys play a significant role in blood pressure regulation via different routes. One important route is the regulation of the renin-angiotensin system (RAS) (Wu et al. 2020a; Paul et al. 2006). Angiotensin-I-converting enzyme (ACE I) participates in the blood pressure regulation process by catalyzing the conversion of angiotensin I to angiotensin II (Ang II) and inactivates bradykinin. Ang II incorporates in hypertension and high levels of blood pressure (Wu et al. 2020b), mediates renal inflammation via NF-κB signaling activation, and improves renal fibrosis via TGF-β1/Smad2/3 pathway (Liu et al. 2012). Subsequently, blocking the catalytic activity of ACE I is a therapeutic target to control hypertension to prevent Ang II accumulation. In addition, the RAS system establishes a balance between angiotensin II (Ang II) and angiotensin (Ang)-(1–7), whereas angiotensin-converting enzyme II (ACE II) prevents the accumulation of Ang II via its conversion to Ang-(1–7). This catalytic reaction is potentiated by bradykinin, which can regulate ACE II (Fernandes et al. 2001). Bradykinin is the principal ligand of bradykinin receptors B1R and B2R. B1R is created and binds to bradykinin as a response to pathophysiologic disorders and could be simulated by inflammation (Leeb-Lundberg et al. 2005). However, the interaction between bradykinin and B2R is regulated by Ang-(1–7) (Fernandes et al. 2001; Leeb-Lundberg et al. 2005). B2R creation is mediated by the activation of mitogen-activated protein kinase (MAPK) pathways (Leeb-Lundberg et al. 2005), enhancing the inflammatory signals via activation of the NF-κB pathway. On the other hand, B2R mediated the RAS route via complexion with ACE II (Fernandes et al. 2001; Leeb-Lundberg et al. 2005). The data showed an enhancement of ACE I activity, associated with suppression of ACE II in the kidney tissues of IRR- and MT-intoxicated animals, and up-regulations of the B1R and B2R protein expressions, which are augmented in the IRR/MT group. However, rutin administration exhibits suppression of ACE I activity, activation of ACE II, and down-regulations of B1R and B2R protein expressions in the kidney tissues of IRR-, MT-, and IRR/MT-intoxicated animals; thus, rutin can prevent Ang II accumulation in the kidney tissues that further regulates renal hypertension. Rutin demonstrates antihypertensive properties in a mechanism comprising ACE I suppression (Oyagbemi et al. 2020).

Micro-RNA has been identified as 20–22 nucleotides arranged in a single non-coding mRNA strand. Micro-RNAs are implicated in normal kidney physiology as well as in kidney pathophysiologic disorders via regulation of their target genes (Salem and Ismail 2021). In the present work, the mRNA gene expression ratio of miR-129–1-3p was down-regulated; however, the relative gene expression ratios of miR-200c-3p and miR-210-3p were up-regulated in the kidney tissues of IRR-, MT-, and IRR/MT-intoxicated rats. The miR-129–1-3p expression exhibited a significant suppressive role of gastric cancer cells via targeting B2R. MiR-129–1-3p was adversely associated with B2R (Wang et al. 2014). However, overexpression of miRNA-200c-3p is a result of Ang II overproduction (Liu et al. 2020) and organizes ACE II protein expression by targeting ACEII 3′ untranslated region (Yu et al. 2021). Consequently, down-regulation of miRNA-200c-3p can counteract the hypertensive nephrotoxicity. On the other hand, miR-210 was overexpressed in damaged renal tubular cells and correlated to HIF-1α overexpression. Down-regulation of miR-210 can attenuate HIF-1α overproduction and preserve renal tubular cells (Feng et al. 2018; Liu et al. 2017). Conversely, rutin treatments ameliorate these miRNAs’ relative gene expression ratios in the kidney tissues of IRR-, MT-, and IRR/MT-intoxicated animals.

## In conclusion

The data showed that rutin administration regulated the gene expression ratios of iNOS, eNOS, and the gene expression ratios of miR-129–1-3p, miR-200c, and miR-210. In addition, it regulated the protein expression ratios of B1R and B2R suppressed ACE I activity and enhanced ACE II activity. Consequently, rutin demonstrated nephroprotection on IRR, MT, and on the combined IRR/MT toxicity in rats due to its potent antioxidant, free radical scavenging, and anti-inflammatory activities, thus, maintaining the kidneys’ structure and function.

## Supplementary Information

Below is the link to the electronic supplementary material.

Supplementary file1 (DOCX 47 KB)

Supplementary file2 (RAR 2726 KB)

## Data Availability

The data will be available upon request.

## Abbreviations

ACE I: Angiotensin-converting enzyme I
ACE II: Angiotensin-converting enzyme II
AchE: Acetylcholinesterase
ALB: Albumin
B1R: Bradykinin receptor I
B2R: Bradykinin receptor II
Ca^2+^: Calcium
CAT: Catalase
CKD: Chronic kidney diseases
CMC: Carboxymethyl cellulose
eNOS: Endothelial nitric oxide synthase
GSH: Reduced glutathione
Gy: Gray
H_2_O_2_: Hydrogen peroxide
HIF-1α: Hypoxia-induced factor–1 alpha
HNO_3_: Nitric acid
H&E: Hematoxylin and eosin
IL-1β: Interleukin–1 beta
IL-2: Interleukin–2
IL-4: Interleukin-4
IL-6: Interleukin 6
iNOS: Inducible nitric oxide synthase
IP: Intraperitoneally
IRR: Ionizing radiation
K^+^: Potassium
MDA: Malondialdehyde
Mg^2+^: Magnesium
miRNA: Micro-RNAs
M-MLV: Employing Moloney murine leukemia virus
MT: Malathion
Na^+^: Sodium
NF-κB: Nuclear factor-kappa B
NO: Nitric oxide
PBS: Phosphate-buffered saline
p-NF-κB p65: Phospho-nuclear factor kappa-light-chain-enhancer of activated B cells p65 subunit
ROS: Reactive oxygen species
RT-PCR: Real-time polymerase chain reaction
SDS: Sodium dodecyl sulfate
SOD: Superoxide dismutase
SPSS: Statistical Package for Social Science
TNF-α: Tumor necrosis factor-alpha
TP: Total-proteins
