# Supplementary material for: Rutin protects against gamma-irradiation and malathion-induced oxidative stress and inflammation through regulation of mir-129-3p, mir-200C-3p, and mir-210 gene expressions in rats’ kidney
Source: Environ Sci Pollut Res Int. 2023 May 15;30(28):72930–48. doi: 10.1007/s11356-023-27166-z (PMC10257612; doi:10.1007/s11356-023-27166-z)
Supplement: Supplementary file 1 — Supplementary file1 (DOCX 47 KB) [file 11356_2023_27166_MOESM1_ESM.docx]

**Protective response of rutin to malathion and gamma-irradiation triggered kidney injury in rats**

**Amel F. M. Ismail^1, *^, Asmaa A. Salem^2^ and Mamdouh M. T. Eassawy^2^**

^1^Drug Radiation Research Department, National Center for Radiation Research and Technology (NCRRT), Egyptian Atomic Energy Authority (EAEA), Cairo, Egypt. *Corresponding Author: [afmismail@gmail.com](mailto:afmismail@gmail.com)

^2^Regional Center for Food and Feed (RCFF), Agricultural Research Center, Giza, Egypt

**Supplementary Data**

**The Structure of Rutin and quercetin**

| 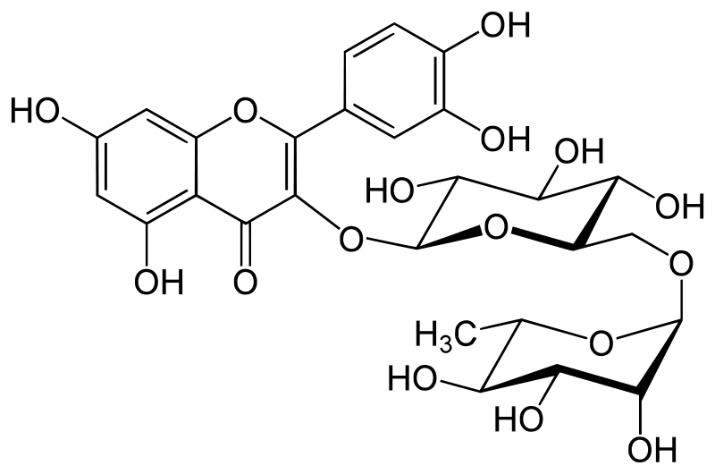  **Rutin** | 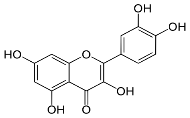  **Quercetin** |
| --- | --- |

Rutin (Quercetin 3 rutinoside or (3,3′,4′,5,7‐pentahydroxyflavone‐3‐rhamnoglucoside) is a naturally occurring flavonoid glycoside (the phenolic compound quercetin is the basic unit of rutin, which is substituted with glucose and rhamnose sugar groups at the hydroxy group in position C-3.

**Materials and methods**

- 1. *Real-time quantitative reverse transcription-polymerase chain reaction*

Total RNA from the frozen kidney pieces was extracted by Qiagen kit (USA), isolated, and inversely transcripted into complementary DNA (cDNA), employing Moloney murine leukemia virus (M-MLV) reverse transcriptase (Promega, Madison, USA). Step One Plus Real-Time PCR System (Applied Biosystems, Foster City, CA, USA) and an SYBR® Green PCR Master Mix (Applied Biosystems) were conducted in a 10 µl final volume, programming the heating cycles: 95°C (10 min), then 40 cycles of 95°C (15 s) and 65 °C (1 min). The sequences of PCR primer pairs and the housekeeping reference gene beta-actin (β-actin) with the corresponding bank gene accession number are denoted in Table 1. The data were evaluated with the ABI Prism sequence detection system software and computed using v1.7 Sequence Detection Software, from PE Biosystems (Foster City, CA). The relative expression values of the studying genes were evaluated using the comparative threshold cycle method. All values were normalized to β-actin, applying the expression 2^-ΔΔCt^ .

Table 1: The sequences of PCR primer pairs and the housekeeping reference gene beta-actin (β-actin) with the corresponding bank gene accession number

| **Gene** | **Primer** | **Sequences（5’→3’）** | **Gene accessions No** |
| --- | --- | --- | --- |
| **β-actin** | Forward | 5′–TCT ACA ATG AGC TGC GTG TG–3' | NC_051347.1 |
|  | Reverse | 5′–TAC ATG GCT GGG GTG TTG AA–3′ |  |
| **iNOS** | Forward | 5′–TCTTTGCTTCTGTGCTAATGCG–3' | NC_051345.1 |
|  | Reverse | 5′–GTTGTTGCTGAACTTCCAATCGT–3' |  |
| **eNOS** | Forward | 5′–TGGGCAGCATCACCTACGATA–3' | NC_051339.1 |
|  | Reverse | 5′–GGAACCACTCCTTTTGATCGAGTTAT–3' |  |

iNOS: inducible nitric oxide synthase, and eNOS: endothelial nitric oxide synthase.
